# Effectiveness of the production of tissue-engineered living bone graft: a comparative study using perfusion and rotating bioreactor systems

**DOI:** 10.1038/s41598-023-41003-w

**Published:** 2023-08-23

**Authors:** Paulina Kazimierczak, Grzegorz Kalisz, Anna Sroka-Bartnicka, Agata Przekora

**Affiliations:** 1 Independent Unit of Tissue Engineering and Regenerative Medicine, Medical University of Lublin, Chodzki 1 Street, 20-093 Lublin, Poland; 2 Independent Unit of Spectroscopy and Chemical Imaging, Medical University of Lublin, Chodzki 4a Street, 20-093 Lublin, Poland

**Keywords:** Biomaterials - cells, Biomedical materials, Biomineralization, Tissues, Adult stem cells, Mesenchymal stem cells

## Abstract

Bioreactor systems are very precious tools to generate living bone grafts in vitro*.* The aim of this study was to compare the effectiveness of rotating and perfusion bioreactor in the production of a living bone construct. Human bone marrow-derived mesenchymal stem cells (BMDSCs) were seeded on the surfaces of hydroxyapatite-based scaffolds and cultured for 21 days in three different conditions: (1) static 3D culture, (2) 3D culture in a perfusion bioreactor, and (3) dynamic 3D culture in a rotating bioreactor. Quantitative evaluation of cell number showed that cultivation in the perfusion bioreactor significantly reduced cell proliferation compared to the rotating bioreactor and static culture. Osteogenic differentiation test demonstrated that BMDSCs cultured in the rotating bioreactor produced significantly greater amount of osteopontin compared to the cells cultured in the perfusion bioreactor. Moreover, Raman spectroscopy showed that cultivation of BMDSCs in the rotating bioreactor enhanced extracellular matrix (ECM) mineralization that was characterized by B-type carbonated substitution of hydroxyapatite (associated with PO_4_^3−^ groups) and higher mineral-to-matrix ratio compared to the ECM of cells cultured in the perfusion system. Thus, it was concluded that the rotating bioreactor was much more effective than the perfusion one in the generation of bone tissue construct in vitro.

## Introduction

Over the years, bone tissue engineering (BTE) has gained increasing interest in clinical applications for the restoration of bone defects. It has been observed that BTE may overcome numerous drawbacks of natural bone grafts (autografts, allografts, and xenografts), such as restricted donor sources, donor-site morbidity, and disease transmission. Application of tissue-engineered bone graft involves the following steps: (i) cell isolation and expansion, (ii) growing of cells on the surface of the scaffold, (iii) in vitro culture of cell-seeded biomaterial to create a living bone graft, and (iiii) implantation of the produced graft into the injury site^1,2^. To successfully create the living bone graft in vitro, the crucial issue is to mimic the in vivo microenvironment by exposing osteoprogenitor cells/mesenchymal stem cells to adequate stimuli/factors. It is well known that the conventional static culture method of three-dimensional (3D) constructs is not good enough to provide appropriate conditions (e.g. sufficient transport of nutrients to the cells) to obtain bone tissue resembling that occurring in vivo. Hence bioreactor systems can be used to improve the robustness and efficiency of bone graft creation by controlling crucial parameters during cell culture and providing homogeneous cell distribution, sufficient gas and nutrient concentrations, waste removal, and mechanical forces^3,4^. There are various types of bioreactor systems, e.g. perfusion bioreactors, rotating bioreactors, spinner flask bioreactors, that can provide suitable conditions for bone graft creation in vitro.

Perfusion bioreactors use a pump system that perfuses culture medium in a continuous way, providing appropriate mass transport of nutrients and gases and controlled mechanical stimuli^5^. Perfusion systems generally consist of a pump, a culture media reservoir, a tubing circuit, vessels that hold the scaffolds, and a waste vessel^3,4^. The important parameter in these systems is the flow rate of the medium that can induce wall shear stresses affecting the microenvironment of cells and thereby supporting the process of bone formation^2,4^. Several studies have shown that cell culture in the perfusion bioreactors enhanced osteogenic differentiation of osteoprogenitor cells/mesenchymal stem cells compared to the static culture by increasing the expression of osteogenesis-related genes (e.g. osteopontin (OPN), bone alkaline phosphatase (bALP), osteocalcin (OC), type I collagen (Col I))^6–8^. In turn, dynamic bioreactors (e.g. rotating bioreactors, spinner flask bioreactors) have been developed primarily to supply nutrients and oxygen in a homogeneous way and create a low-shear environment which plays an important role in improving osteogenesis by activating mechanotransduction signaling pathways^6,9^. The National Aeronautics and Space Administration (NASA)-developed Rotary Cell Culture System (RCCS) simulates relative microgravity conditions, providing both a low-shear environment and optimal mass transfer^9^. The most common RCCS consists of a rotary base with control of the rotational speed and a horizontally rotating vessel. The cell-seeded biomaterials may be maintained either in a free-fall manner or fixed on a needle in the rotating vessel. Moreover, this type of bioreactor may also be used without biomaterials to generate 3D cell aggregates resembling tissues in vivo^2^. Similar to perfusion bioreactors, some studies have shown that the rotation-based cultivation method increased the expression of osteogenesis-related genes (e.g. bALP, OC, Col I) and extracellular matrix (ECM) mineralization in mesenchymal stem cells compared to the static culture^10–12^.

Another crucial aspect that should be taken into account when creating the living bone graft in vitro is the selection of a suitable scaffold material that serves as a platform for the expansion of bone-forming cells. Importantly, 3D cell culture on the surface of the scaffold provides conditions that partially mimic in vivo microenvironment as it allows for appropriate cell–cell cross-talk, intercellular signaling network, and cell-extracellular matrix components interactions^13^. Therefore, it is crucial that biomaterial used for the living bone graft generation is characterized by high biocompatibility, osteoconductivity, and ideally osteoinductivity. Scaffold possessing all mentioned features supports cell adhesion, migration, proliferation, and osteogenic differentiation. Moreover, bone scaffolds for BTE applications should possess a macroporous microstructure with interconnected pore networks to facilitate cell ingrowth and provide the space for the formation of new blood vessels after implantation. High macroporosity of the biomaterial also ensures uniform diffusion of nutrients, metabolites, and gases within the implant. Another important feature of the bone scaffold is its slow biodegradability supporting the natural bone formation and remodeling in vivo^2,3,14,15^. It is also recommended that the production method of scaffolding biomaterial is cost-effective^15^.

The development of a cost-effective and efficient production method of bone graft in vitro for clinical applications is a big challenge for BTE. There are a number of commercially available artificial tissue-engineered bone allografts e.g. INFUSE^®^ (Medtronic Spinal and Biologics, Memphis, TN, USA), Osteocel^®^ Plus, Osteocel^®^ PRO (Nuvasive, San Diego, CA, USA), CeLLogix (Omnia Medical, Morgantown, WV, USA), Trinity ELITE^®^ (Orthofix Medical Inc., Lewisville, TX, USA)^16^. However, the production costs of most of them are high which makes the value of the bone grafts and substitutes market (involving allografts, bone grafts substitutes, cell-based matrices) to be estimated at $2586.30 million in 2021, and is projected to reach $4005.26 million by 2031^17^. According to the available literature, many studies have reported a biological evaluation of bioengineered bone tissue construct after culture in one type of bioreactor system^6–8,10–12^. Nevertheless, since the effectiveness of the formation of bone construct in vitro highly depends on the culture conditions, it is justified to compare various bioreactor systems that are most often used for BTE purposes.

Thus, the aim of this study was to compare the effectiveness of perfusion and rotating bioreactor systems in the creation of the living bone graft in vitro. In the research the Lazar Arrow-MTM Micro Bioreactor System (Lazar Research Laboratories, Inc., Los Angeles, CA, USA) and Rotary Cell Culture System (RCCS, Synthecon, Houston, TX, USA) were used as model perfusion and rotating system, respectively. There is a great diversity of dynamic 3D bioreactors for BTE applications, however various bioreactor systems differ in terms of cost-effectiveness, simplicity to use, monitoring options, productivity, scale of production, and recommended applications. The Lazar Arrow-MTM Micro Bioreactor System belongs to easy to use perfusion bioreactors with continuous medium flow in an open-loop system and with indirect perfusion (the medium flows around the cell-seeded biomaterial). In turn, the RCCS is a unique 3D cell culture technology with the ability to stimulate relative microgravity conditions. It is frequently used for the generation of 3D cellular and tissue models for various applications. The selected for the purposes of this study bioreactors are simple to operate and low-cost devices designed to work on a laboratory scale. Nevertheless, unlike typical bioreactors used on an industrial scale, control of crucial cultivation parameters, such as pH, gas and nutrient concentration in the medium, and shear stress is impossible due to the simplicity of these devices. On the other hand, although bioreactors used in the industry for biopharmaceutical, food, agricultural, and biotechnological applications have the capability to control crucial conditions for large-scale production, they are very specialized and technologically advanced devices, generating high costs of the production process. Nevertheless, in this study, constant cell culture conditions were ensured by placing both bioreactors (perfusion and rotating) and static culture (in a multiwell plate) in a 37 ˚C incubator with a humidified atmosphere of 95% air and 5% CO_2_. Therefore all tested groups were subjected to the same environmental conditions (i.e. temperature, humidity, CO_2_ concentration and environmental disruption due to opening/closing the bioreactor door). To compare the effectiveness of selected perfusion and rotating bioreactor systems in the generation of the living bone graft, bone marrow-derived mesenchymal stem cells (BMDSCs) and developed previously hydroxyapatite-based bone scaffold with proven high biocompatibility, osteoconductivity, and osteoinductivity^18–20^, were used. Selected for these comparative studies model biomaterial is made of nanopowder of hydroxyapatite and chitosan/agarose matrix. Importantly, the biomaterial is characterized by high bioactivity (ability to form apatite crystals on their surface), biodegradability, rough microstructure, high open (70%) and interconnected macroporosity, compressive strength comparable to cancellous bone, and carries a low risk of inflammatory response^18–20^, indicating that it is a good platform for the expansion of bone-forming cells during the creation of a living bone graft in vitro. The use of the bone scaffold with all above-mentioned features ensured good adhesion and proliferation of BMDSCs and was a guarantee of the success of these comparative studies. Moreover, in this study, 3D culture of BMDSCs in static conditions was performed and it served as a test control. Cell proliferation, differentiation, and mineralization were compared to select 3D culture conditions that provide the most favorable microenvironment for cell cultivation, supporting the generation of the bone construct in the most efficient way.

## Methods

### Bone scaffold fabrication

**Figure 1:**
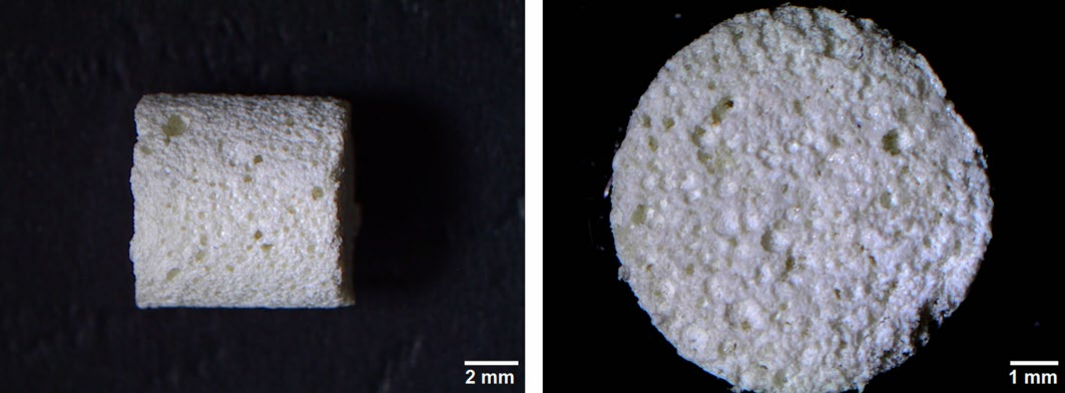
Microstructure of the fabricated scaffold visualized by a stereoscopic microscope.

Highly macroporous biomaterials acting as a platform for cell growth were produced in accordance with the procedure described in the Polish Patent no. 235822 and with the method described previously^19,21^. Briefly, 2% (*w/v*) chitosan (50–190 kDa MW, Sigma-Aldrich Chemicals, Warsaw, Poland), 5% (*w/v*) agarose (gel point 36 ± 1.5 °C, Sigma-Aldrich Chemicals, Warsaw, Poland), and 40% (*w/v*) hydroxyapatite nanopowder (particle size < 200 nm, Sigma-Aldrich Chemicals, Warsaw, Poland) were suspended in acetic acid solution (Avantor Performance Materials, Gliwice, Poland) and mixed. Subsequently, sodium bicarbonate (Sigma-Aldrich Chemicals, Warsaw, Poland) was added. The obtained paste was transferred into cylinder-shaped forms and subjected to heating (95 °C), cooling, freezing, and then freeze-drying. Finally, the resultant biomaterials were immersed in sodium hydroxide solution (Avantor Performance Materials, Gliwice, Poland), washed with deionized water, and air-dried. The microstructure of the fabricated scaffold was visualized by a stereoscopic microscope (Olympus SZ61TR, Olympus Polska Sp. z o. o., Warsaw, Poland (Fig. 1). Prior to cell culture experiments, the scaffolds were sterilized using ethylene oxide.

### Cell culture

The study was performed with the use of human bone marrow-derived mesenchymal stem cells (BMDSCs) purchased from American Type Culture Collection Cell Bank (ATCC-LGC Standards, Teddington, UK). BMDSCs were cultured in a Mesenchymal Stem Cell Basal Medium (ATCC-LGC Standards, Teddington, UK) supplemented with the components of Bone Marrow-Mesenchymal Stem Cell Growth Kit (ATCC-LGC Standards, Teddington, UK) and antibiotics (10 U/mL penicillin and 10 µg/mL). Cells were maintained at 37 °C in a humidified atmosphere with 95% air and 5% CO_2._

### Static and dynamic 3D cultures

**Figure 2:**
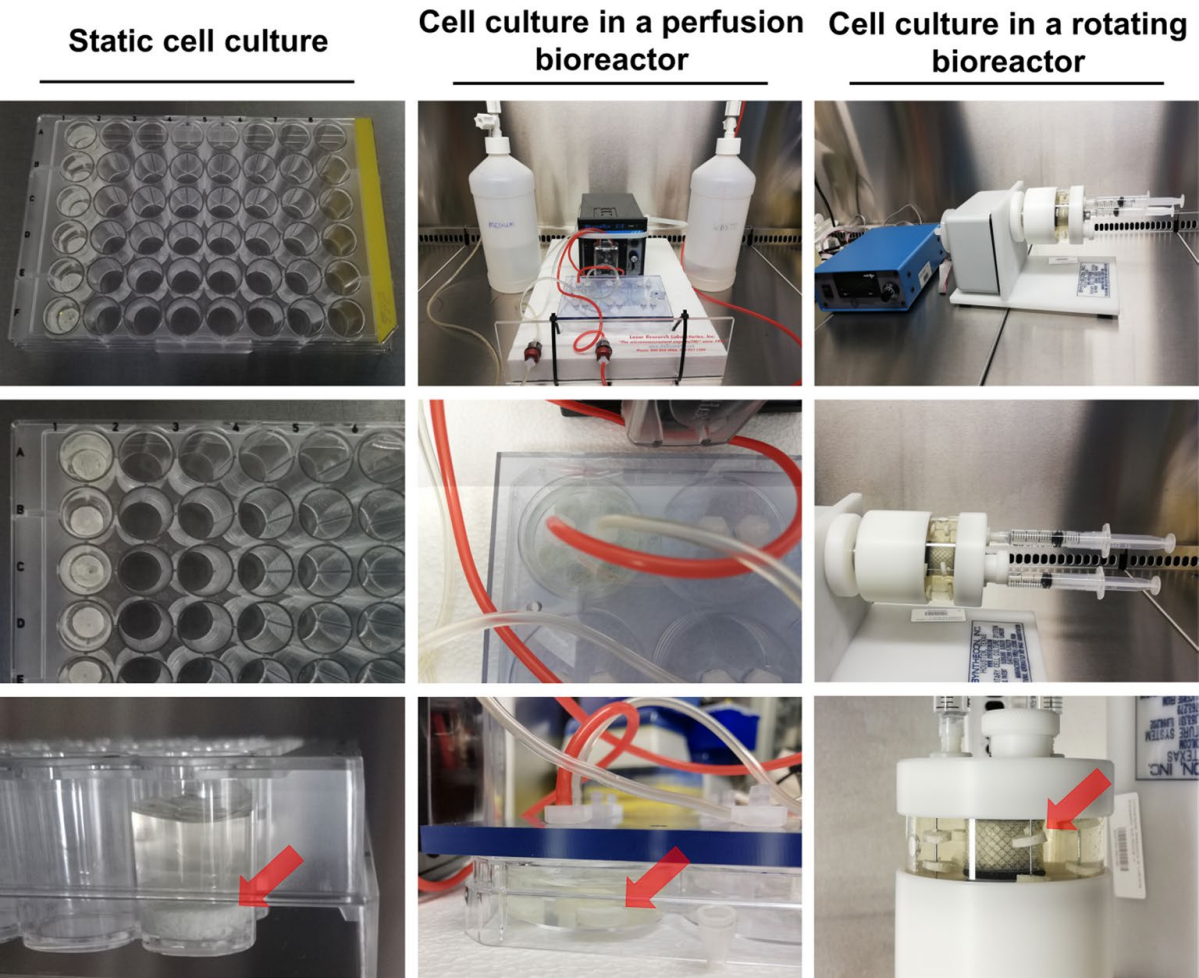
Setup of 3D BMDSC cultures in the static conditions, in the perfusion bioreactor (Lazar Arrow-MTM Micro Bioreactor System), and in the rotating bioreactor (Rotary Cell Culture System (RCCS), Synthecon). Red arrows indicate the placement of cell-seeded biomaterials.

BMDSCs (passage 2) were seeded directly on the surface of biomaterials (8 mm in diameter and 3 mm thick) placed in the wells of the 48-multiwell plate in 500 µL of complete culture medium at a density of 1 × 10^5^ cells/mL. Before placing the biomaterials into bioreactors (24 h after seeding), cell viability on the scaffolds was checked using a Live/Dead Double Staining Kit (Sigma-Aldrich Chemicals, Warsaw, Poland) in accordance with the manufacturer’s method. Stained cells were observed using a confocal laser scanning microscope (CLSM, Olympus Fluoview with FV1000, Olympus Polska Sp. z o. o., Warsaw, Poland). Next, part of the cell-seeded biomaterials was left in the wells of the 48-multiwell plate and treated as a static 3D culture (samples marked as mat_S), whereas the remaining part of the cell-seeded biomaterials was put in the perfusion bioreactor (Lazar Arrow-MTM Micro Bioreactor System, Lazar Research Laboratories, Inc., Los Angeles, CA, USA) (samples marked as mat_P) and in the rotating bioreactor (Rotary Cell Culture System (RCCS), Synthecon, Houston, TX, USA) (samples marked as mat_R) with a continuous rotation rate equal to 1 rpm. Figure 2 demonstrates the representative setup of static and dynamic 3D cultures. 3D cell cultures in the static conditions and in the perfusion bioreactor were maintained in the independent wells of the plate. In the case of the rotating bioreactor, cell-seeded biomaterials were fixed on the needles and were cultured in the same rotating vessel. The setup of static and dynamic 3D cell cultures was carried out three times as three independent experiments.

To induce osteogenic differentiation of BMDSCs, cells were cultured in the osteogenic medium made of complete culture medium supplemented with 10 mM β-glycerophosphate, 50 µg/mL ascorbic acid, and 10^−7^ M dexamethasone (Sigma-Aldrich Chemicals, Warsaw, Poland). The osteogenic medium was added to the cells after 24-h culture on the surface of the biomaterials in a complete culture medium. Next, the static cell culture and bioreactor setups were transferred to the incubator and maintained at 37 °C in a humidified atmosphere with 95% air and 5% CO_2_. In the case of static cell culture, cells were cultured in 500 µL of osteogenic medium and every fourth day, half of the medium was replaced with a fresh portion. In turn, BMDSCs maintained in the perfusion bioreactor were cultured on the scaffolds placed in the wells of a 6-multiwell plate in 5 mL of osteogenic medium with a perfusion speed (medium flow rate) of 20 µL/min. In the rotating bioreactor, cell-seeded biomaterials were fixed on a needle that is linked with a base of the rotating vessel containing 50 mL of the osteogenic medium. Every fourth day, 10 mL of the medium was replaced with a fresh portion. After 21 days of static and dynamic 3D cultures, biomaterials were subjected to various analyses described in the following subsections.

### Evaluation of cell growth and morphology on the biomaterials

Evaluation of cell growth and morphology on the surface of biomaterials 24 h after seeding and after long-term culture (21 days) in the static and dynamic conditions was performed by the cytoskeleton and nuclei staining with AlexaFluor635-conjugated phallotoxin and DAPI, respectively. The cells were fixed with paraformaldehyde, permeabilized with Triton X-100, blocked with bovine serum albumin (all reagents from Sigma-Aldrich Chemicals, Warsaw, Poland), and stained with AlexaFluor635-conjugated phallotoxin (Invitrogen, Carlsbad, California, USA) and DAPI (Sigma-Aldrich Chemicals, Warsaw, Poland) in accordance with the method described previously^22^. The stained cells were observed using the CLSM and obtained images were analyzed using ImageJ software version 1.52a to count cell nuclei. Additionally, visualization of cell-seeded biomaterials was also performed by a scanning electron microscope (SEM, JEOL JCM-6000Plus, Japan). For SEM visualization, the samples were prepared by fixation with paraformaldehyde and dehydration in graded ethanol. Dehydrated samples were next sputtered with a thin layer of gold. Detailed protocol for sample preparation was described previously^23^.

### Evaluation of cell proliferation

Cell proliferation was evaluated via measurement of the content of total cytoplasmic lactate dehydrogenase (LDH) after cell lysis using the Lactate Dehydrogenase Activity Assay Kit (Sigma-Aldrich Chemicals, Warsaw, Poland). To determine cell number increase with time, the assay was performed at two time intervals: (1) 24 h after cell seeding and (2) after 21 days of culture. The assay was carried out based on the manufacturer’s procedure. According to the protocol, the content of total LDH detected after cell lysis is proportional to the number of cells in a population. Thus, the higher OD values were detected at 490 nm with the reference wavelength of 690 nm, the greater number of BMDSCs were present on the biomaterials.

### Osteogenic differentiation assessment

To evaluate the osteogenic differentiation process of BMDSCs, osteogenic markers (type I collagen (Col I), osteopontin (OPN), bone sialoprotein 2 (BSP2), osteocalcin (OC)) were determined in cell lysates using appropriate enzyme-linked immunosorbent assays (ELISAs) (EIAab ELISAs kit, Wuhan, China). The cell lysates were obtained by two freeze–thaw cycles and a sonication process (ultrasonic processor UO100H Hielscher Ultrasound Technology, Teltow, Germany) for 30 s at 30% amplitude. The level of osteogenic markers was normalized to the total cellular protein content, which was estimated for each sample using a BCA Protein Assay Kit (Thermo Fisher Scientific, Waltham, Massachusetts, USA). The ELISA results were expressed both as ng of osteogenic maker per mL (referred to as original ELISA results) and as ng of osteogenic marker per mg of total cellular proteins (normalized ELISA data). Osteogenic differentiation was also determined by immunofluorescent (IF) staining of osteogenic markers (Col I and osteonectin (ON)). Samples for IF staining were prepared in accordance with the procedure described previously^22^.

### ECM mineralization assessment by Raman spectroscopy and chemometric analysis

Raman spectroscopy of samples was performed using a DXR Raman Microscope (Thermo Scientific, Waltham, MA, USA). The device was equipped with a laser of 780 nm excitation wave and output power of 15 mW. For obtaining the best Raman Intensity of recorded spectra, parameters of measurement were optimized in the spectral range of 900–1000 cm^−1^ with 10 × objective and CCD camera. A 50-pinhole aperture was used. For spectra analysis, 30 measurements were recorded with dedicated software (Omnic ver. 8.2.0.387, Thermo Fisher Scientific, Madison, WI, USA). Spectral analysis, Principal Component Analysis (PCA), and hyperspectral analysis were performed with Orange hyperspectral data processing suite, with Quasar software (ver. 1.5.0). The chosen preprocessing steps were spectral range selection for the 200–2000 cm^−1^, the Savitzky Golay smoothing using 2 points algorithm, and normalization to the 960 cm^−1^ band.

Different ratios of Raman peaks were used to visualize spatial bone maturation. For each pixel, color shows the ratio of those two peaks at that pixel with a heatmap scale with fixed values of min–max between groups. The CytoSpec software (ver. 2.00.01, Berlin, Germany) was used for performing these analyses.

### Statistical analysis

Experiments were performed at least in triplicate (n ≥ 3). The obtained data were displayed as mean values ± SD. Statistically significant results between samples were considered at *P* < 0.05 using one-way ANOVA followed by Tukey's test (GraphPad Prism 8.0.0 Software).

## Results

### Cell growth and proliferation on the bone scaffolds after culture in the static and dynamic conditions

**Figure 3:**
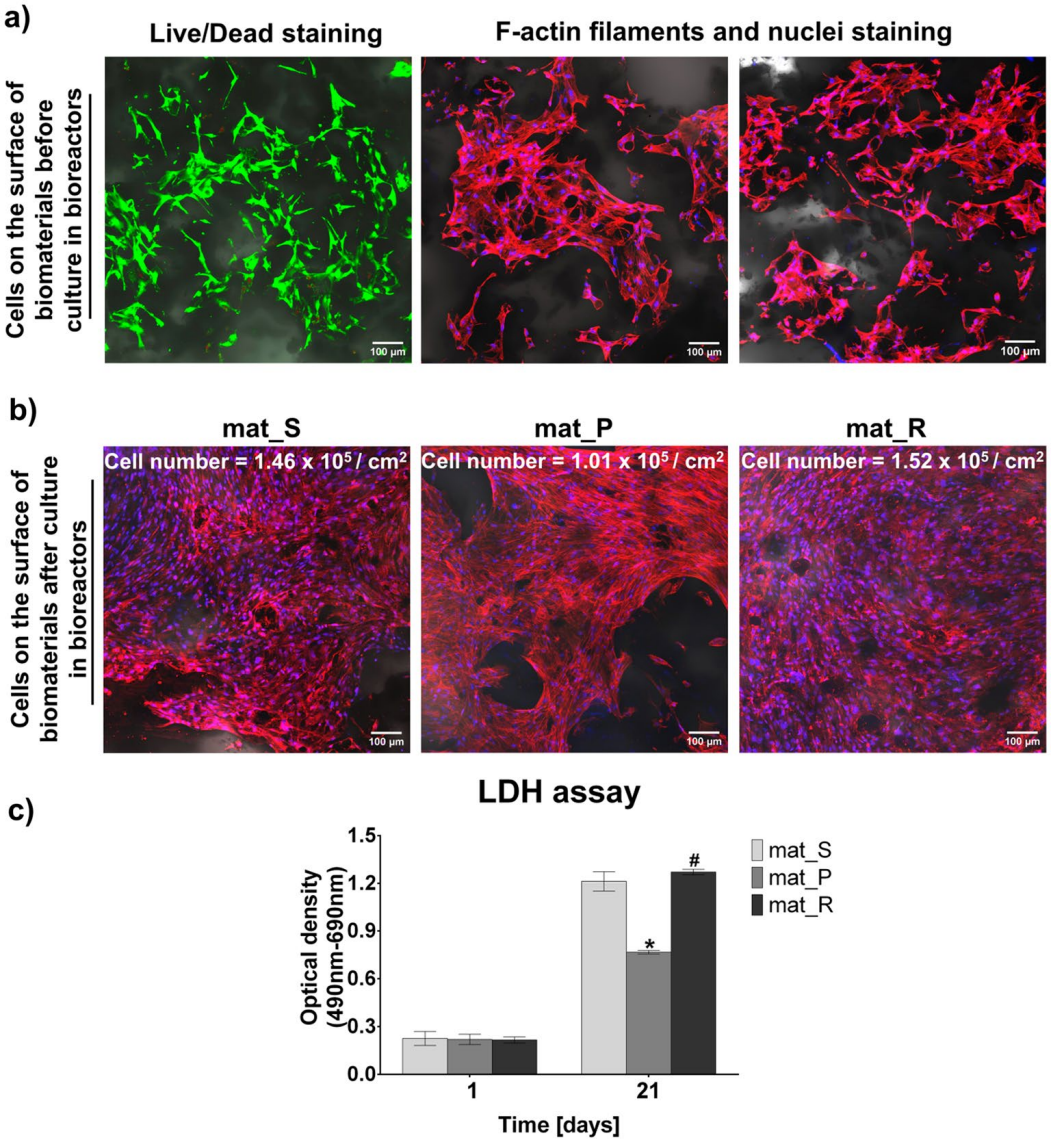
Evaluation of viability, morphology, and proliferation of BMDSCs: (**a**) CLSM images of BMDSCs on the surface of bone scaffolds before culture in the bioreactors (Live/Dead staining: green fluorescence indicates viable cells, red fluorescence indicates dead cells; F-actin filaments and DAPI staining: red fluorescence indicates cytoskeleton filaments, blue fluorescence indicates nuclei; magnification × 100); (**b**) CLSM images of BMDSCs on the surface of bone scaffolds after 21 days of culture in the static and dynamic conditions (red fluorescence indicates cytoskeleton filaments, blue fluorescence indicates nuclei; cell number was evaluated by nuclei counting per 1 cm^2^ of biomaterial surface using ImageJ software version 1.52a; magnification × 100); (**c**) cell proliferation assessment by total LDH assay (*statistically significant results compared to mat_S, ^#^statistically significant results compared to mat_P, *P* < 0.05, one-way ANOVA followed by Tukey’s test).

Figure 3 shows the evaluation of cell viability, adhesion, morphology, and number before and after 21 days of culture in static and dynamic conditions. Before placing cell-seeded biomaterials into the bioreactors, cell viability and morphology were determined by Live/Dead and F-actin filaments staining, respectively (Fig. 3a). Confocal laser scanning microscope (CLSM) images showed a great number of viable cells (green fluorescence) on the surfaces of the bone scaffolds 24 h after seeding. Moreover, F-actin filaments staining showed that BMDSCs were well spread and had flattened morphology indicating their good adhesion and growth on the surface of biomaterials. After 21 days of culture, CLSM images showed that cells cultured on the surface of biomaterials in the rotating bioreactor (mat_R) reached a full confluence, whereas cells maintained in the perfusion bioreactor (mat_P) reached approx. 70% confluence. In turn, BMDSCs after culture in the static condition (mat_S) showed approx. 90% confluence (Fig. 3b). Thus, obtained results prove that the surface of biomaterials supported cell proliferation regardless of the applied culture conditions. This observation was confirmed by quantitative evaluation of cell number that was performed by total LDH assay and nuclei counting using ImageJ software. The analysis demonstrated that the number of BMDSCs on the surface of biomaterials after culture in the perfusion bioreactor (mat_P) was significantly lower (*P* < 0.0001) compared to the static culture (mat_S) and culture in the rotating bioreactor (mat_R) (Fig. 3b,c).

**Figure 4:**
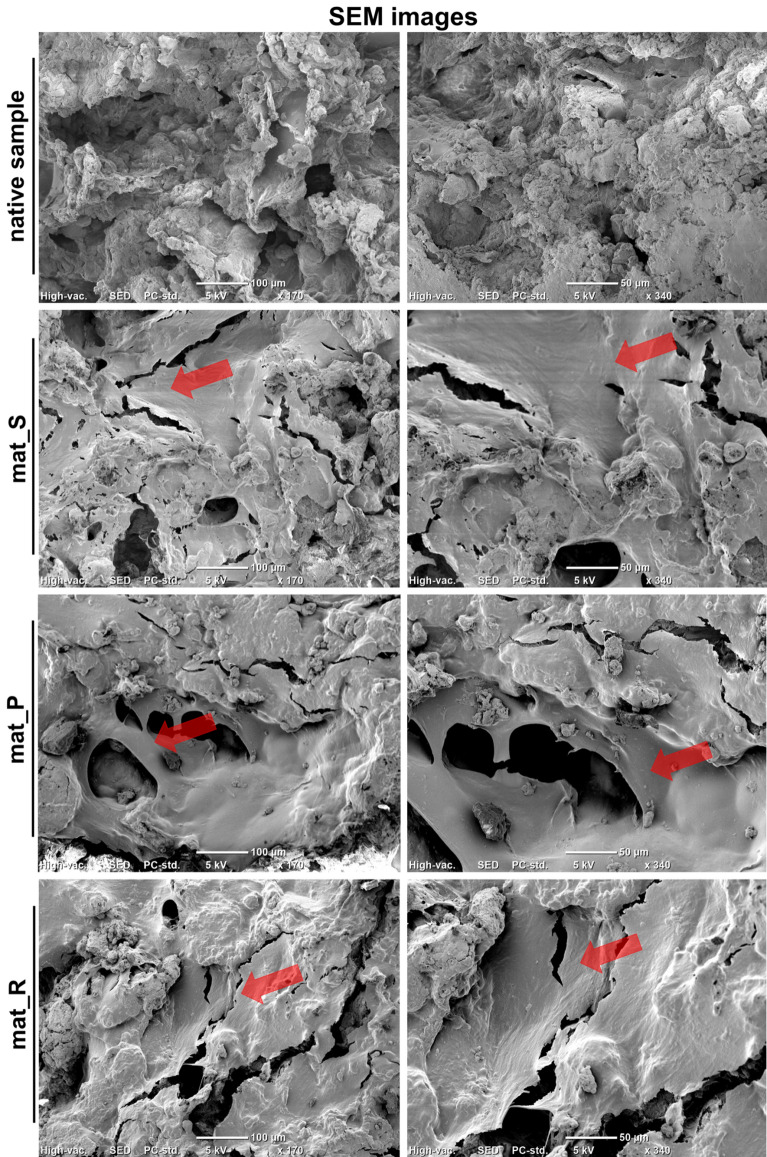
SEM images presenting BMDSCs on the surface of bone scaffolds after 21-day static and dynamic 3D cultures (red arrows indicate sheets of the cells; magnification × 170 and × 340).

Staining of cell cytoskeleton (F-actin filaments) after 21-day culture showed relatively uniform cell distribution on the surface of bone scaffolds, even after culture in the static conditions (Fig. 3b). The obtained results are consistent with the scanning electron microscope (SEM) imaging that demonstrated large sheets of BMDSCs on the surfaces of biomaterials after 21 days of culture regardless of applied conditions (static or dynamic) (Fig. 4). Moreover, cell infiltration into pores of the biomaterials was observed.

### BMDSCs osteogenic differentiation assessment

**Figure 5:**
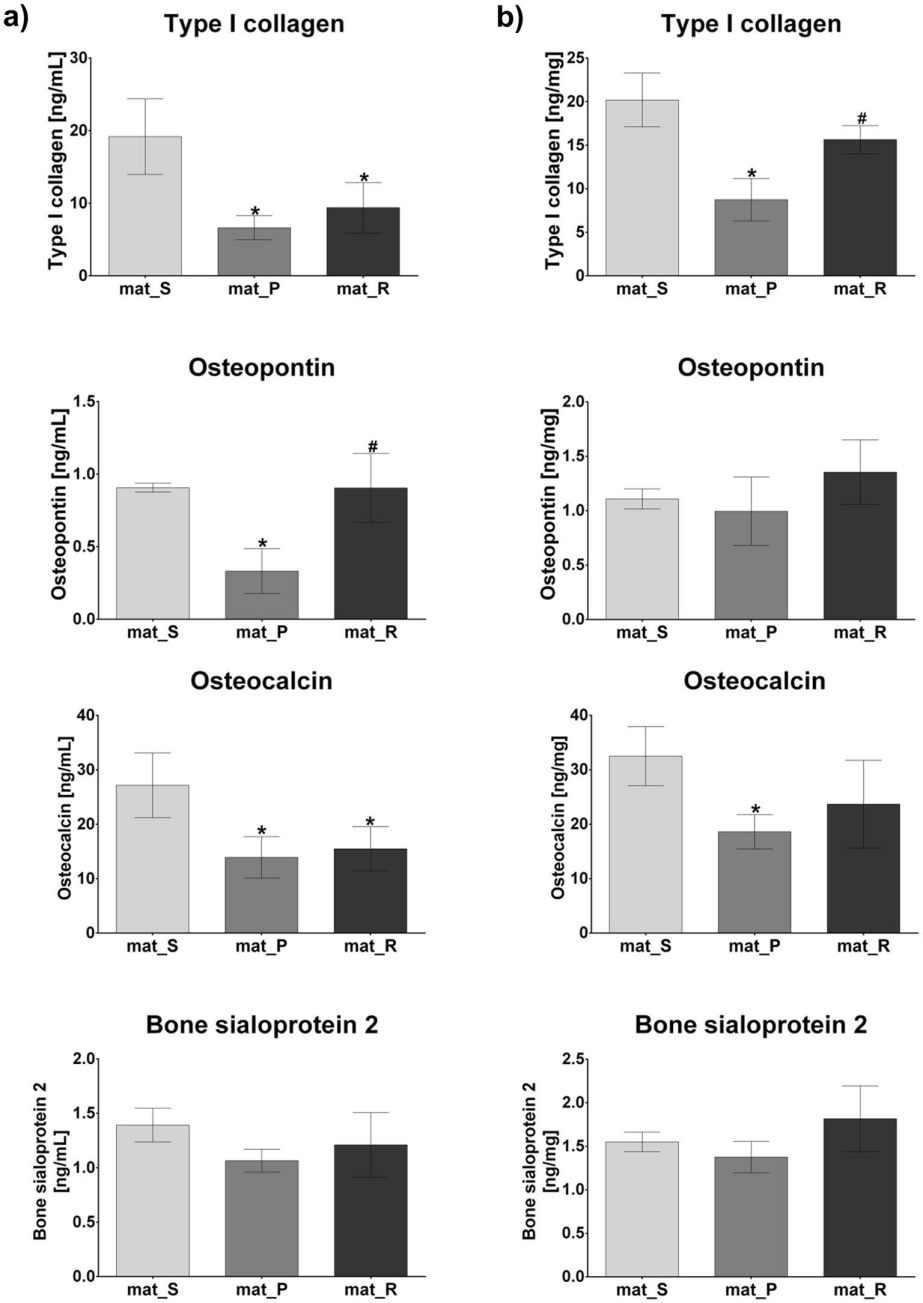
The level of osteogenic markers determined by ELISAs in BMDSCs after 21-day static and dynamic 3D cultures. The results were expressed as (**a**) ng of marker per mL (original ELISA data) and as (**b**) ng of marker per mg of total cellular proteins (normalized ELISA data); *statistically significant results compared to mat_S, ^#^statistically significant results compared to mat_P, *P* < 0.05, one-way ANOVA followed by Tukey’s test.

The level of osteogenic markers was determined by ELISAs in BMDSCs after 21-day static and dynamic 3D cultures. Figure 5a presents original (raw) ELISA results (expressed as ng of marker per mL), whereas Fig. 5b shows normalized data per mg of total cellular proteins. Normalization was performed to check whether enhanced proliferation and thus higher cell biomass on the sample maintained in the rotating bioreactor directly affected the results of osteogenic differentiation assay. Interestingly, the trend of obtained results was kept unchanged after normalization, namely the level of osteogenic markers was higher for mat_R compared to mat_P regardless of the presentation of the results (with normalization or original ELISA data). It confirms that rotating bioreactor supported osteogenic differentiation process to a greater extent than perfusion one. However, after normalization fewer statistically significant differences between the samples were observed. Generally, BMDSCs cultured in the static conditions (mat_S) produced higher amounts of osteogenic markers as compared to the cells grown in both bioreactors (mat_P and mat_R) (Fig. 5). Original ELISA results showed that despite a similar amount of cells that were present on the surfaces of biomaterials after 21 days of culture in the static conditions and in the rotating bioreactor, the production of osteogenic markers (Col I with *P* < 0.05 and OC with *P* < 0.05) was significantly higher in BMDSCs cultured in a stationary way (Fig. 5a). Nevertheless, normalized ELISA data did not reveal statistically significant differences in the level of osteogenic markers between mat_S and mat_R (Fig. 5b). Considering results without normalization per mg of total proteins, BMDSCs cultured in the rotating bioreactor (mat_R) produced comparable amounts of Col I, BSP2, and OC to the cells maintained in the perfusion system (mat_P). However, it was observed that 3D culture in the rotating bioreactor resulted in significantly greater amounts of OPN (*P* < 0.01) (Fig. 5a). Moreover, the level of OPN for mat_R was comparable to that for mat_S. After normalization of the results, cells grown in the rotating system still produced greater amount of OPN than BMDSCs cultured in the perfusion bioreactor, however obtained results were not statistically significant (Fig. 5b). Nevertheless, normalization revealed significantly greater amount of Col I (*P* < 0.01) for mat_R sample compared to mat_P.

**Figure 6:**
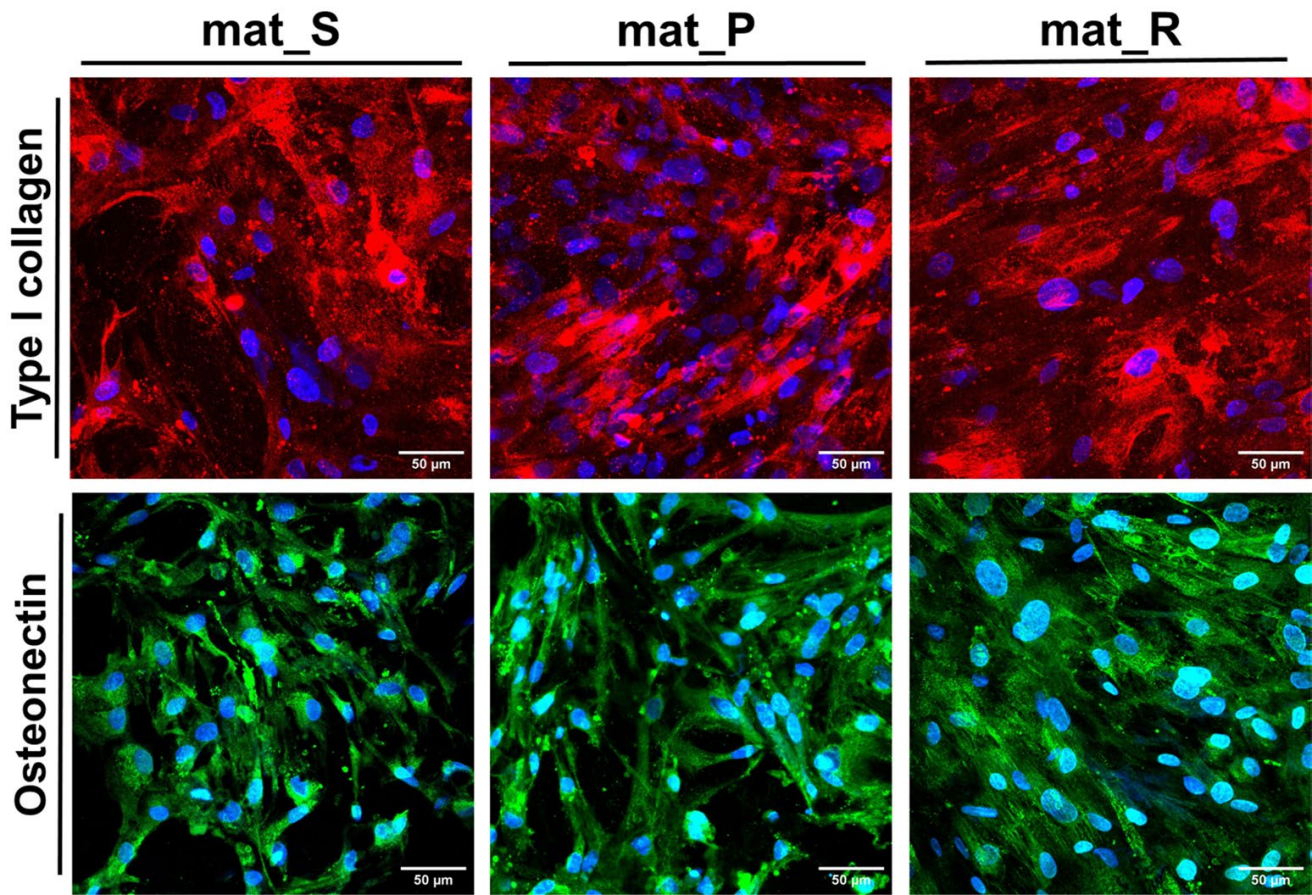
CLSM images presenting immunofluorescent staining of type I collagen and osteonectin in the extracellular matrix of BMDSCs after 21-day static and dynamic 3D cultures (red fluorescence indicates type I collagen; green fluorescence indicates osteonectin, blue fluorescence indicates nuclei; magnification × 200).

CLSM images of selected osteogenic markers stained by IF technique did not reveal noticeable differences in the amounts of Col I and ON in the ECM of BMDSCs cultured in the bioreactor systems compared to the static conditions (Fig. 6).

### ECM mineralization assessment by Raman spectroscopy and chemometric analysis

**Figure 7:**
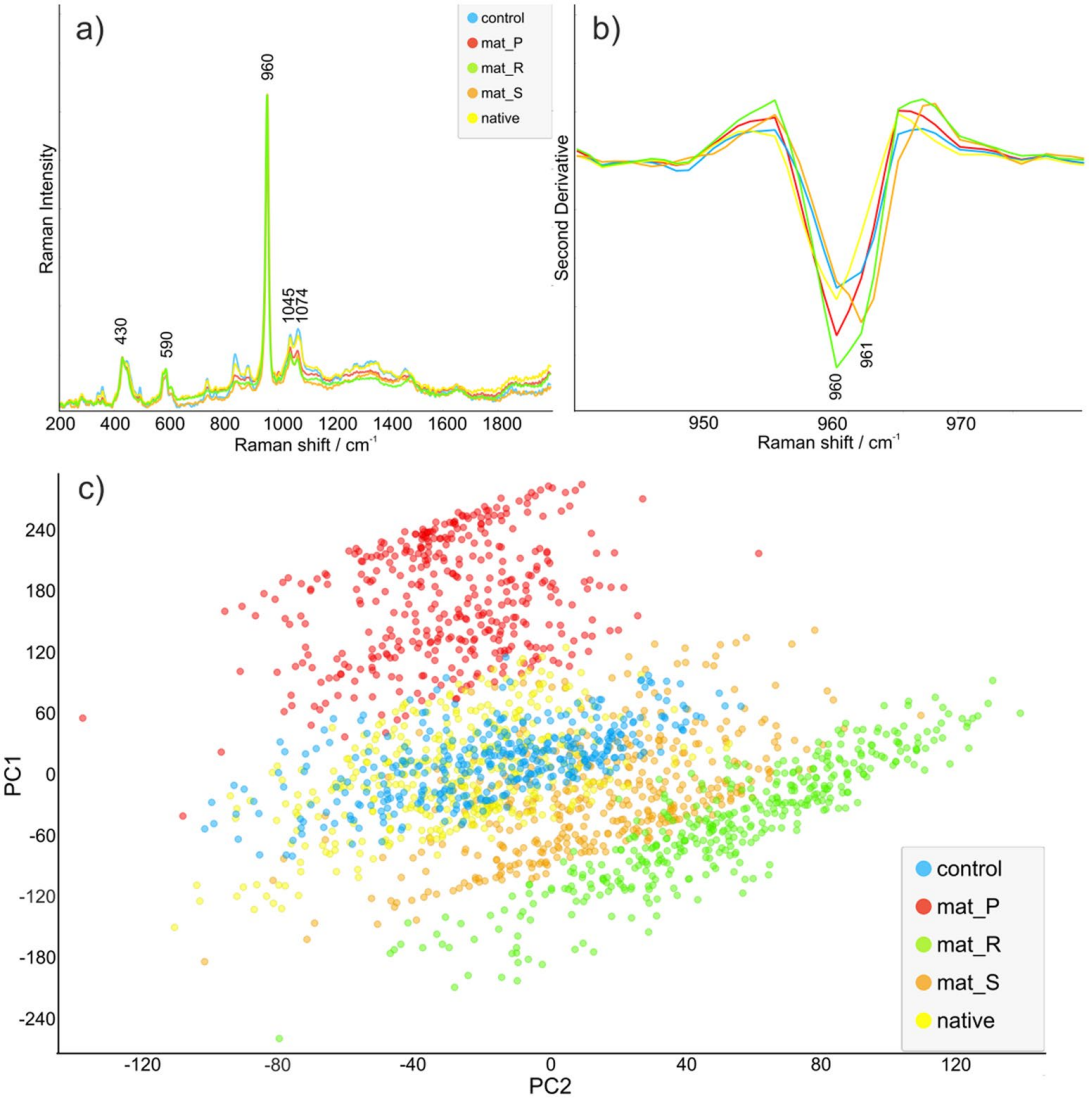
Raman analysis of cell-seeded biomaterials after 21-day static and dynamic 3D cultures: (**a**) Raman mean spectra for investigated samples normalized to 960 cm^−1^; (**b**) second derivative of Raman spectra in range 940–980 cm^−1^; (**c**) a principal component analysis (PCA) score plot of control and investigated samples with 83% of variance (native—untreated biomaterial, control—non-seeded biomaterial incubated in the culture medium).

The resulting mean spectra for investigated samples are presented in Fig. 7a. For better comparison spectra were normalized to the highest band at 960 cm^−1^, assigned to ν_1_ stretching of P–O bands in hydroxyapatite crystals’ PO_4_ groups^24^. Spectra had characteristic features ascribed to hydroxyapatite, with band maxima at 430, 590, 960, 1045, and 1074 cm^−1^ corresponding to phosphate group vibrations: ν_2_, ν_4_, ν_1_, ν_3,_ and (CO_3_)^2−^, respectively^25^. The untreated biomaterial (native) was the reference sample for cell-seeded biomaterials maintained in static conditions (mat_S), in bioreactors (mat_P and mat_R) and for non-seeded biomaterial incubated in the culture medium (sample marked as control). Full spectra of native, control and mat_S seemed identical. Transformation to amorphous calcium phosphate (ACP) was not observed in spectra, due to the lack of 960 band shifting to 950 cm^−1^ in average spectra^24^. However, the second derivative revealed in mat_S (Fig. 7b) a small shift from 960 to 961 cm^−1^ that can be assigned to a less crystalline form of hydroxyapatite^26^. It is noteworthy that mat_R had lower intensity in the range of 1200–1600 cm^−1^, corresponding to the organic components of biogenic hydroxyapatite, compared to all other groups^27^.

To compare the variance of investigated materials, Principal Component Analysis (PCA) was performed in datasets of 400 spectra/group. The scatter plot of PC1 vs. PC2 is shown in Fig. 7c, calculated for Raman spectra. It can be seen that there was an overlap between control and native samples, and good separation of spectra was observed in direction of PC1, which accounted for 83% of the variance. Clearly, mat_P and mat_R were located in different regions of the score plot. The resulting PCA data was in agreement with averaged spectra, differing by range 1012–1096 cm^−1^ assigned to ν_3_ vibrations of a phosphate group.

According to a validated method by Taylor et al. peak area ratio of ν_1_ PO_4_/δ CH_2_ was considered a Raman mineral/matrix ratio (MMR)^28^. The calculated results of MMR and other ratios are shown in Table 1. MMR did not differ between native (10.52174) and control (10.07191) samples. Decrease of mineralization was observed in mat_S (8.783927) and mat_P (9.79339) but increase was noted in mat_R (14.53026). The decrease in MMR values detected for mat_S and mat_P compared to the native (untreated) and control (non-seeded, incubated in culture medium) samples was most likely the result of the presence of a great number of cells on the scaffolds that produced ECM proteins. Whereas native and control hydroxyapatite-based biomaterials were not covered by organic components in the form of cells and ECM proteins, so MMR values for these samples were higher due to the presence of mineral constituent on the surface. In turn, a higher MMR value calculated for mat_R compared to the native and control samples despite the presence of a great number of cells on its surface (as it was proven in cell growth and proliferation assays, Figs. 3b,c and Fig. 4) is strong evidence for enhanced mineralization of the 3D culture in the rotating bioreactor. Further hydroxyapatite spectral evaluation was performed by band intensity ratios in relation to the main phosphate lattices. 430 and 590 to 960 cm^−1^ were investigated, but the results resembled control and the ones acquired from bioreactors. However, results acquired from the intensity of ν_3_ to ν_1_ and ν_1_ CO_3_ B-substitution differed significantly between samples. Carbonate substitution (CO_3_^2−^) is one of the important processes in bone mineral and bone metabolism, dependent on the age of individuals^29^. Type B is related to natural bones in their early phase and type A is more common in older ones^30^. Typical bands for type B were reported to fluctuate between 1065–1075 cm^−1^ and type A in the range^31^ 1095–1103 cm^−1^. Improved biological performance of the CO_3_^2−^ substituted hydroxyapatite can make the bone grafts more prone to remodeling after implantation^32^. To assess the differences between the samples, ratios of selected bands were used.

**Table 1:** Raman intensity ratio calculated for phosphate vibrations, B-substitution and mineral-to-matrix (native—untreated biomaterial, control—non-seeded biomaterial incubated in the culture medium).

	ν_2_ PO_4_/ν_1_ PO_4_	ν_4_ PO_4_/ν_1_ PO_4_	ν_3_ PO_4_/ν_1_ PO_4_	HA/B-type carbonate	ν_1_ CO_3_^2−^/ν_1_ PO_4_	Mineral/matrix ratio (MMR)
	I_430_:I_960_	I_590_:I_960_	I_1045_:I_960_	I_960_:I_1070_	I_1074:_I_1045_	I_960_:I_1446_
Native	0.168329	0.121071	0.219678	4.457572	1.038316	10.52174
Control	0.17	0.116149	0.21799	4.885875	1.028165	10.07191
mat_S	0.176087	0.131632	0.192998	5.215009	1.020776	8.783927
mat_P	0.168193	0.120087	0.187137	5.333321	0.96386	9.79339
mat_R	0.167115	0.126833	0.173639	7.473819	0.883923	14.53026

**Figure 8:**
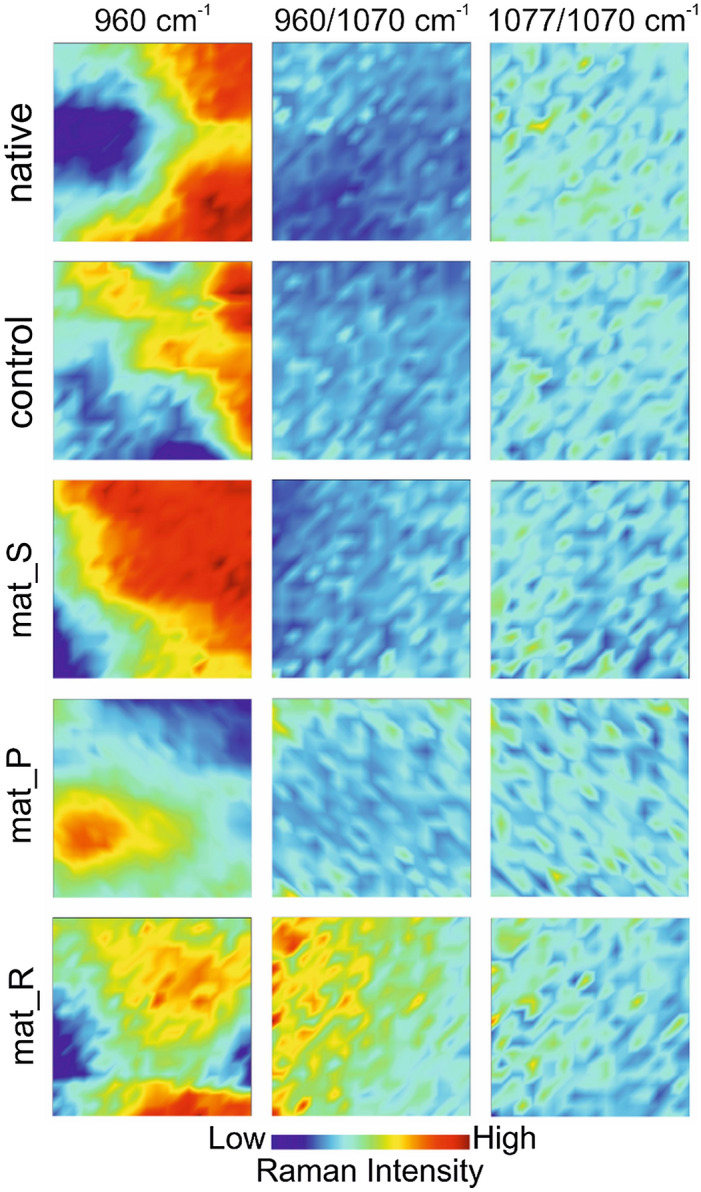
Raman distribution images of ν_1_ PO_3_^4−^ peak (960 cm^−1^), ratio phosphate HA/carbonated HA (960/1070 cm^−1^), and ratio of total HA/carbonated HA (1077/1070 cm^−1^) (native—untreated biomaterial, control—non-seeded biomaterial incubated in the culture medium).

The maturation of the mineral in samples was compared with ratio distribution indicated by intensity changes in the phosphate (1077 and 960 cm^−1^) and carbonate (1070 cm^−1^) bands and is presented in Fig. 8 with fixed ranges for better comparison between samples^33^. Phosphate to B-type carbonate distribution (I_1077_:I_1070_) was evenly distributed in all investigated groups. Interestingly, total hydroxyapatite to B-type carbonate (I_960_:I_1070_) revealed increased substitution in mat_R and slightly in mat_P, revealing a newly formed bone-like structure^31,33^.

## Discussion

The main role of the skeleton is to bear the body loads, therefore it is subjected to constant mechanical stimulation. Dynamic bone loading and emerging mechanical stimuli are important factors in the bone regeneration process in vivo^34^. In BTE, bioreactors are very valuable dynamic-based systems for imitating the microenvironment of natural bone under in vitro conditions since they may control and provide appropriate parameters during cell culture by ensuring mechanical stimuli. Moreover, bioreactor systems are crucial tools to generate ex vivo bone tissue constructs by culturing autologous bone-forming cells on the surface of 3D porous biomaterials^3^. In this study, BMDSCs were cultured on the surface of previously developed hydroxyapatite-based scaffolds with microstructural and mechanical features comparable to cancellous bone^19^. Three different conditions were applied: (1) static 3D cell culture (mat_S), (2) 3D culture in the perfusion bioreactor with flow rate of 20 μL/min (mat_P), and (3) in the rotating bioreactor with rotation rate of 1 rpm (mat_R). This approach allowed the selection of the most effective 3D culture conditions to generate the living bone graft in vitro.

Within these studies it was demonstrated that culture of mesenchymal stem cells in the rotating bioreactor resulted in significantly greater (*P* < 0.0001) proliferation compared to the perfusion system (Fig. 3b,c). It is an important issue since the appropriate cell number is crucial to ensure sufficient cell biomass for osteogenic differentiation and bone formation process^35^. Obtained results are consistent with the studies by Yamada et al.^8^ who reported that during the direct perfusion (1.6 mL/min flow rate), proliferation of rat BMDSCs on polyester-based scaffolds was significantly inhibited. Similarly, Salifu et al.^6^ proved that human fetal osteoblasts (hFOB 1.19 cell line) cultured on the surface of the polycaprolactone/hydroxyapatite scaffold in the perfusion system (direct perfusion with a flow rate of 1.6 mL/min) showed reduced proliferation. Interestingly, Mitra et al.^36^, revealed that indirect perfusion culture (media volume in the bioreactor was 12 mL; 3 mL/min flow rate) of BMDSCs seeded onto hydroxyapatite/poly(lactide-*co*-glycolide) scaffold increased cell number. It should be noted that observed divergence of reports in the available literature regarding the effect of cultivation in perfusion bioreactors on cell proliferation may result from the various flow rates, culture chambers, type of perfusion (direct or indirect), origin of the stem cells, volume and type of the culture medium and scaffolds used. In this study the applied flow rate in the perfusion bioreactor was very low (20 μL/min) compared to the systems described in the literature (from 0.1 mL/min to even 10 mL/min)^7,8,37,38^. Moreover, the cells were cultured in a small volume of the medium (5 mL) in an open-loop system, whereas other authors used either direct perfusion or higher volume of the culture medium or closed-loop system. Applied in this research volume of the culture medium and flow rate were selected experimentally during pilot studies to provide the highest cell viability and growth rate. Type of the scaffold used for cell cultivation has also great impact on the final results. For instance Yamada et al. used microporous scaffold^8^, whereas in this study scaffold characterized by high macroporosity was used. Notably, it was observed that BMDSCs had the ability to grow into the interconnected pore network of the scaffolds, which is an important phenomenon for the generation of bone tissue construct in vitro since it facilitates natural bone formation^3^. All mentioned differences in the experimental setup make it difficult to compare obtained results with the reports by other authors.

BMDSCs have been considered as one of the most suitable cell sources for BTE due to their good osteogenic differentiation capacity^39^. The standard procedure for the induction of osteogenic differentiation of mesenchymal stem cells in vitro is cell culture in the presence of dexamethasone (a synthetic glucocorticoid), ascorbic acid, and β-glycerophosphate^40^. Osteogenic differentiation is a multistage and complex process whose each step is characterized by typical markers: (1) proliferative phase, (2) ECM synthesis and maturation, and (3) ECM mineralization. At the first step of osteogenic differentiation, cells actively proliferate to ensure sufficient cell biomass for osteogenic differentiation and bone formation process and produce primarily Col I and fibronectin. In the first stage, cells also exhibit low bALP activity, low to moderate OPN production, and express Runt-related transcription factor 2 (RUNX2). Next, there is decreased proliferation rate and cells start to express bALP activity at a high level, which provides phosphates during the ECM mineralization process. At the last stage, a high expression of OC, OPN, ON, and BSPs is observed, followed by calcium and phosphate deposition (ECM mineralization phase)^41–43^.

In this study, the level of typical osteogenic markers (Col I, OPN, BSP2, and OC) produced by BMDSCs grown on the surface of the biomaterials under static and dynamic conditions was evaluated after 21-day culture by ELISAs (Fig. 5). It was observed that cells cultured in the static conditions (mat_S) produced higher amounts of osteogenic markers as compared to the cells grown in bioreactors (mat_P and mat_R). Interestingly, the obtained results of this study are not consistent with the reports in the available literature regarding the increase in the production of osteogenic markers by mesenchymal stem cells after culture in bioreactors^6–8,10,11^. It was assumed that greater production of osteogenic markers in 3D cultures maintained in the static conditions resulted from a small volume (500 µL) of the culture medium that contained more concentrated growth factors and cytokines (autocrine and paracrine signals) responsible for promotion of osteogenic differentiation. In contrast, mat_P was cultured in 5 mL of osteogenic medium with a medium flow rate of 20 µL/min, whereas mat_R was cultured in 50 mL osteogenic medium. Frequency of the medium renewal could also have affected osteogenic differentiation. In the case of mat_S and mat_R the medium renewal occurred every fourth day, which was sufficient to maintain high viability of the BMDSCs thanks to their culture on 3D and highly porous samples (lag phase, known as adaptation phase, is longer and proliferation rate is slower on 3D scaffolds compared to 2D culture). It was half portion (250 µL) for mat_S and 10 mL (out of 50 mL) for mat_R. Whereas continuous flow of the medium in the perfusion system resulted in the renewal of 28.8 mL of the medium each day, so the frequency was as high as 5.76 times a day. It is well known that the degree of osteogenic differentiation of mesenchymal stem cells highly depends on a level of intrinsic and extrinsic factors, including surrounding cells (paracrine regulation) and signals secreted by the cells themselves (autocrine regulation)^44^. Thus, maintenance of the 3D culture in a small amount of culture medium increased the concentration of crucial molecules responsible for the promotion of osteogenic differentiation in the surrounding microenvironment. However, the main aim of this study was to compare the effectiveness of two commonly used bioreactor systems (perfusion bioreactor—Lazar Arrow-MTM Micro Bioreactor System and rotating bioreactor—RCCS, Synthecon) in the formation of the bone graft in vitro, whereas 3D culture in the static conditions served only as a test control. Comparative evaluation of these two bioreactor systems indicated that the rotating system was more supportive for osteogenic differentiation than the perfusion one since it led to significantly greater (*P* < 0.01) OPN production (original ELISA data) and (*P* < 0.05) Col I synthesis (normalized ELISA data). Interestingly, Gandhi et al.^7^ reported that rat BMDSCs encapsulated within fibrin beads and cultured in the perfusion bioreactor with a flow rate of 10 mL/min exhibited increased expression of OPN. The OPN is a non-collagenous protein and is responsible for calcium binding and the proper process of ECM mineralization^41^. Thus, it is an important osteogenic marker in the formation of new mineralized bone tissue in vivo and a promising predictor during bone graft production in vitro. Osteogenic differentiation of BMDSCs cultured in the static and dynamic conditions was also determined by Col I and ON staining using the IF technique (Fig. 6). Col I is the most abundant collagenous protein in ECM of bones and plays a pivotal role in determining bone strength. Whereas ON (also known as SPARC) is a late marker of osteogenic differentiation (the mineralization phase) and belongs to the non-collagenous proteins regulating calcium release by binding collagen and hydroxyapatite crystals, thus affecting collagen mineralization during bone formation^45^. Importantly, performed IF staining of ECM proteins showed that BMDSCs cultured on the surface of hydroxyapatite-based scaffolds in both static and dynamic conditions formed an expanded 3D network of ECM, that plays a pivotal role in the regulation of cell adhesion, proliferation, and differentiation in vivo^45^. Thus, a living bone graft with an evolved ECM microstructure, which was created under in vitro conditions in this study, may enhance the formation of new bone at the site of the fracture, supporting the repair and regeneration of the damaged bone.

Raman spectroscopy is widely used in research on biominerals and biomedicine providing structural insight on microscopic level. Particularly its ability to evaluate both mineral and organic matter simultaneously is considered beneficial in bone-related research^46–48^. Hydroxyapatite phase transformation and carbonate substitution are possible to investigate on micro- and nano-scale with modern Raman devices in various aspects, due to minimal preparation needed^49,50^. Natural hydroxyapatite is a non-stoichiometric structure containing carbonate groups built into the structure. Carbonate ions decrease crystallinity and crystal growth and increase the solubility of hydroxyapatite in an acidic environment which is naturally created by active osteoclasts, thus enhancing hydroxyapatite biodegradation^46,51^. According to the crystal position carbonate, apatite is divided into type A and type B (associated with OH^−^ and PO_4_^3−^ groups, respectively)^31^. Overall, Raman measurements of mineral and organic matrix allowed comparison of ECM mineralization in cell-seeded biomaterials maintained in static and dynamic conditions. The direct transformation to amorphous calcium phosphate was not observed, but B-type carbonated substitution of hydroxyapatite and higher mineral-to-matrix ratio in the mat_R clearly indicated that the rotating bioreactor provided more efficient conditions for the fabrication of bone construct in vitro.

## Conclusions

BTE tools may overcome many of the limitations and complications associated with the clinical application of natural bone grafts. However, the effective formation of living bone graft in vitro is still a big challenge for BTE. In this study, a comparative analysis of different 3D culture conditions using perfusion and rotating bioreactors was presented to select the most effective system that provides favorable microenvironment for cell cultivation and supports the generation of the bone construct in vitro. Biological assessment and Raman analysis of cell-seeded biomaterials after 21 days of culture showed that the rotating bioreactor (Rotary Cell Culture System, Synthecon) is a better tool to produce tissue-engineered bone grafts for regenerative medicine applications than the perfusion bioreactor (Lazar Arrow-MTM Micro Bioreactor System) since it exerted a superior effect on the cellular response, for example by supporting BMDSCs proliferation, ECM synthesis and mineralization. Importantly, mineralized ECM was characterized by B-type carbonated substitution of hydroxyapatite (associated with PO_4_^3−^ groups) and higher mineral-to-matrix ratio, revealing a newly formed bone-like structure. Thus, rotating system provided not only greater cellular biomass on the bone scaffold, but also promoted formation of well-mineralized bone-like structures. Interestingly, according to original (raw) ELISA results, cells cultured in the static conditions (that served only as a test control) produced higher amounts of osteogenic markers as compared to the cells grown in both bioreactors. Nevertheless, normalization of the ELISA results to the total cellular proteins did not reveal statistically significant differences between scaffolds cultured in the static conditions and in the rotating system. Moreover, it should be noted that the cultivation of cell-seeded biomaterials in the rotating bioreactor allowed the cells to overgrow the entire surface of the biomaterial, boosting the bone-forming capacity of tissue-engineered constructs. In contrast, culture of the cells in the static conditions allowed formation of the bone-like structure only on the top surface of the biomaterial.

## Data Availability

The datasets generated during and/or analysed during the current study are available in the Mendeley Data repository, 10.17632/xj8mpsdt6k1^52^.
